# Supplementary material for: A simple method for unsupervised anomaly detection: An application to Web time series data
Source: PLoS One. 2022 Jan 11;17(1):e0262463. doi: 10.1371/journal.pone.0262463 (PMC8752013; doi:10.1371/journal.pone.0262463)
Supplement: S1 Appendix — (PDF) [file pone.0262463.s001.pdf]

S1 Appendix State space representation

In this appendix, we show the state space representation of the models in the evaluation and application sections.

S1A Model in the evaluation section

The observation model can be represented as follows.

$$Y_n = \boldsymbol{F}_n \boldsymbol{X}_n + \epsilon_{Y,n} \;\; \epsilon_{Y,n} \sim \mathcal{N}(0, \sigma_Y^2)$$

where

$$\begin{aligned} \boldsymbol{F}_n &= \left[\begin{array}{c|cccc|c|cc} 1 & 1 & 0 & \cdots & 0 & \boldsymbol{D}_n & 1 & 0 \end{array} \right] \\ \boldsymbol{X}_n &= \left[\begin{array}{c|cccc|c|cc} T_n & S_n & S_{n-1} & \cdots & S_{n-22} & \boldsymbol{h}_n & A_n & a_2 A_{n-1} \end{array} \right]^{\mathrm{T}} \end{aligned}$$

The vector $\boldsymbol{F}_n$ is 1×49 vector and $\boldsymbol{D}_n = [D_{1,n}, D_{2,n}, \cdots, D_{23,n}]$. The vector $\boldsymbol{X}_n$ is 49×1 vector and $\boldsymbol{h}_n = [h_{1,n}, h_{2,n}, \cdots, h_{23,n}]^{\mathrm{T}}$.

The system model can be represented as follows.

$$\boldsymbol{X}_n = \boldsymbol{G}\boldsymbol{X}_{n-1} + \boldsymbol{\omega}_n, \;\; \boldsymbol{\omega}_n \sim \mathcal{N}(\boldsymbol{0}, \boldsymbol{W})$$

where

$$
\boldsymbol{G} = \left[\begin{array}{c|cccc|c|cc}
1 & 0 & 0 & \cdots & 0 & \mathbf{0} & 0 & 0 \\ \hline
0 & -1 & -1 & \cdots & -1 & \mathbf{0} & 0 & 0 \\
0 & 1 & 0 & \cdots & 0 & \mathbf{0} & 0 & 0 \\
0 & 0 & \ddots & 0 & 0 & \mathbf{0} & 0 & 0 \\
0 & 0 & 0 & 1 & 0 & \mathbf{0} & 0 & 0 \\ \hline
0 & 0 & 0 & \cdots & 0 & \boldsymbol{I}_{(23\times 23)} & 0 & 0 \\ \hline
0 & 0 & 0 & \cdots & 0 & \mathbf{0} & a_1 & 1 \\
0 & 0 & 0 & \cdots & 0 & \mathbf{0} & a_2 & 0
\end{array}\right]
$$

$$
\boldsymbol{\omega}_n = \left[\begin{array}{c|cccc|c|cc} \omega_{T,n} & \omega_{S,n} & 0 & \cdots & 0 & \boldsymbol{\omega}_{H,n} & \omega_{A,n} & 0 \end{array}\right]^{\mathrm{T}}
$$

$$
\boldsymbol{W} = \mathrm{diag}\left(\begin{array}{c|cccc|ccc|cc} \sigma_T^2 & \sigma_S^2 & 0 & \cdots & 0 & \sigma_H^2 & \cdots & \sigma_H^2 & \sigma_A^2 & 0 \end{array}\right)
$$

The matrix $\boldsymbol{G}$ is 49×49 matrix and $\boldsymbol{I}_{(23\times 23)}$ indicates 23×23 identity matrix. The vector $\boldsymbol{\omega}_n$ is 49×1 vector and $\boldsymbol{\omega}_{H,n} = [\omega_{H,1,n}, \omega_{H,2,n}, \cdots, \omega_{H,23,n}]$. The matrix $\boldsymbol{W}$ is 49×49 diagonal matrix.

S1B Model in the application section

The observation model can be formulated as follows.

$$
\boldsymbol{Y}_n = \boldsymbol{F}_n \boldsymbol{X}_n + \boldsymbol{\epsilon}_n, \;\; \boldsymbol{\epsilon}_n \sim \mathcal{N}(\mathbf{0}, \boldsymbol{V})
$$

where

$$
\boldsymbol{Y}_n = \begin{bmatrix} Y_{1,n} \\ Y_{2,n} \end{bmatrix}, \boldsymbol{F}_n = \left[\begin{array}{cc} 1 & 0 \\ 0 & 1 \\ \hline \boldsymbol{C}_O & \boldsymbol{0} \\ \boldsymbol{0} & \boldsymbol{C}_O \\ \hline h_n & 0 \\ 0 & h_n \\ \hline \boldsymbol{E}_n & \boldsymbol{0} \\ \boldsymbol{0} & \boldsymbol{E}_n \\ \hline \boldsymbol{Z}_n & \boldsymbol{0} \\ \boldsymbol{0} & \boldsymbol{Z}_n \\ \hline 1 & 0 \\ 0 & 0 \\ 0 & 1 \\ 0 & 0 \end{array}\right]^{\mathrm{T}}, \boldsymbol{X}_n = \left[\begin{array}{c} T_{1,n} \\ T_{2,n} \\ \hline \boldsymbol{D}_{1,n-5:n} \\ \boldsymbol{D}_{2,n-5:n} \\ \hline H_{1,n} \\ H_{2,n} \\ \hline \boldsymbol{s}_{1,n} \\ \boldsymbol{s}_{2,n} \\ \hline \boldsymbol{p}_{1,n} \\ \boldsymbol{p}_{2,n} \\ \hline A_{1,n} \\ a_{1,2}A_{1,n-1} \\ A_{2,n} \\ a_{2,2}A_{2,n-1} \end{array}\right], \boldsymbol{\epsilon}_n = \begin{bmatrix} \epsilon_{1,n} \\ \epsilon_{2,n} \end{bmatrix}, \boldsymbol{V} = \begin{bmatrix} \sigma^2_{Y_1} & \sigma^2_{Y_1Y_2} \\ \sigma^2_{Y_1Y_2} & \sigma^2_{Y_2} \end{bmatrix}
$$

In the matrix $\boldsymbol{F}_n$, 2×34 matrix, $\boldsymbol{C}_O = [1\ 0 \cdots 0]^{\mathrm{T}}$, h_n is a holiday dummy which takes 1 if day n is a holiday and takes 0 otherwise, $\boldsymbol{E}_n = [E_{1,n}, E_{2,n}, E_{3,n}]^{\mathrm{T}}$ is a season dummy vector, and $\boldsymbol{Z}_n = [Z_{1,n}, Z_{2,n}, Z_{3,n}, Z_{4,n}]^{\mathrm{T}}$ is an advertising expenditure vector. In the vector $\boldsymbol{X}_n$, 34×1 vector, $\boldsymbol{D}_{i,n-5:n} = [D_{i,n}, D_{i,n-1}, \cdots, D_{i,n-5}]^{\mathrm{T}}$, $\boldsymbol{s}_{i,n} = [s_{i,1,n}, s_{i,2,n}, s_{i,3,n}]^{\mathrm{T}}$ is a season component vector, and $\boldsymbol{p}_{i,n} = [p_{i,1,n}, p_{i,2,n}, p_{i,3,n}, p_{i,4,n}]^{\mathrm{T}}$ is an advertising component vector for the time series i.

The system model can be represented as follows.

$$
\boldsymbol{X}_n = \boldsymbol{G}\boldsymbol{X}_{n-1} + \boldsymbol{\omega}_n, \ \ \boldsymbol{\omega}_n \sim \mathcal{N}(\boldsymbol{0}, \boldsymbol{W})
$$

where

$$
\boldsymbol{G} = \left[\begin{array}{cc|cc|cc|cc|cc|cccc}
1 & 0 & 0 & 0 & 0 & 0 & 0 & 0 & 0 & 0 & 0 & 0 & 0 & 0 \\
0 & 1 & 0 & 0 & 0 & 0 & 0 & 0 & 0 & 0 & 0 & 0 & 0 & 0 \\
\hline
0 & 0 & \boldsymbol{C}_S & \boldsymbol{0} & 0 & 0 & 0 & 0 & 0 & 0 & 0 & 0 & 0 & 0 \\
0 & 0 & \boldsymbol{0} & \boldsymbol{C}_S & 0 & 0 & 0 & 0 & 0 & 0 & 0 & 0 & 0 & 0 \\
\hline
0 & 0 & 0 & 0 & 1 & 0 & 0 & 0 & 0 & 0 & 0 & 0 & 0 & 0 \\
0 & 0 & 0 & 0 & 0 & 1 & 0 & 0 & 0 & 0 & 0 & 0 & 0 & 0 \\
\hline
0 & 0 & 0 & 0 & 0 & 0 & \boldsymbol{I}_{(3\times 3)} & \boldsymbol{0} & 0 & 0 & 0 & 0 & 0 & 0 \\
0 & 0 & 0 & 0 & 0 & 0 & \boldsymbol{0} & \boldsymbol{I}_{(3\times 3)} & 0 & 0 & 0 & 0 & 0 & 0 \\
\hline
0 & 0 & 0 & 0 & 0 & 0 & 0 & 0 & \boldsymbol{I}_{(4\times 4)} & \boldsymbol{0} & 0 & 0 & 0 & 0 \\
0 & 0 & 0 & 0 & 0 & 0 & 0 & 0 & \boldsymbol{0} & \boldsymbol{I}_{(4\times 4)} & 0 & 0 & 0 & 0 \\
\hline
0 & 0 & 0 & 0 & 0 & 0 & 0 & 0 & 0 & 0 & a_{1,1} & 1 & 0 & 0 \\
0 & 0 & 0 & 0 & 0 & 0 & 0 & 0 & 0 & 0 & a_{1,2} & 0 & 0 & 0 \\
0 & 0 & 0 & 0 & 0 & 0 & 0 & 0 & 0 & 0 & 0 & 0 & a_{2,1} & 1 \\
0 & 0 & 0 & 0 & 0 & 0 & 0 & 0 & 0 & 0 & 0 & 0 & a_{2,2} & 0
\end{array}\right]
$$

$$
\boldsymbol{\omega}_n = \left[\begin{array}{cc|cc|cc|cc|cc|cccc}
\omega_{T_1,n} & \omega_{T_2,n} & \boldsymbol{\omega}_{D_1} & \boldsymbol{\omega}_{D_2} & \omega_{H_1,n} & \omega_{H_2,n} & \boldsymbol{\omega}_{S_1,n} & \boldsymbol{\omega}_{S_2,n} & \boldsymbol{\omega}_{P_1,n} & \boldsymbol{\omega}_{P_2,n} & \omega_{A_1,n} & 0 & \omega_{A_2,n} & 0
\end{array}\right]^{\mathrm{T}}
$$

$$
\boldsymbol{W} = \mathrm{diag}\left(\begin{array}{cc|cc|cc|cc|cc|cccc}
\sigma^2_{T_1} & \sigma^2_{T_2} & \boldsymbol{\sigma}^2_{D_1} & \boldsymbol{\sigma}^2_{D_2} & \sigma^2_{H_1} & \sigma^2_{H_2} & \boldsymbol{\sigma}^2_{S_1} & \boldsymbol{\sigma}^2_{S_2} & \boldsymbol{\sigma}^2_{P_1} & \boldsymbol{\sigma}^2_{P_2} & \sigma^2_{A_1} & 0 & \sigma^2_{A_2} & 0
\end{array}\right)
$$

In the matrix $\boldsymbol{G}$, 34×34 matrix, $\boldsymbol{C}_S$ is represented as follows.

$$
\boldsymbol{C}_S = \begin{bmatrix}
-1 & -1 & \cdots & -1 \\
1 & 0 & \cdots & 0 \\
0 & \ddots & 0 & 0 \\
0 & 0 & 1 & 0
\end{bmatrix}
$$

In the vector $\boldsymbol{\omega}_n$, 34×1 vector, $\boldsymbol{\omega}_{D_i} = [\omega_{D_i,n} \ \ 0 \ \cdots \ 0]$, $\boldsymbol{\omega}_{S_i,n} = [\omega_{S_i,1,n}, \omega_{S_i,2,n}, \omega_{S_i,3,n}]$, and $\boldsymbol{\omega}_{P_i,n} = [\omega_{P_i,1,n}, \omega_{P_i,2,n}, \omega_{P_i,3,n}, \omega_{P_i,4,n}]$ for the time series i. In the matrix $\boldsymbol{W}$, 34×34 matrix, $\boldsymbol{\sigma}^2_{D_i} = [\sigma^2_{D_i} \ \ 0 \ \cdots \ 0]$,

$\boldsymbol{\sigma}^2_{S_i} = [\sigma^2_{S_i,1}, \sigma^2_{S_i,2}, \sigma^2_{S_i,3}]$, and $\boldsymbol{\sigma}^2_{P_i} = [\sigma^2_{P_i,1}, \sigma^2_{P_i,2}, \sigma^2_{P_i,3}, \sigma^2_{P_i,4}]$ for the time series i.
