# Supplementary material for: A simple method for unsupervised anomaly detection: An application to Web time series data
Source: PLoS One. 2022 Jan 11;17(1):e0262463. doi: 10.1371/journal.pone.0262463 (PMC8752013; doi:10.1371/journal.pone.0262463)
Supplement: S2 Table — We show the optimal threshold, F1 score, precision, and recall on 59 time series in the A1 benchmark under each case when we apply the local level model. If the F1 score is undefined under any k, we enter NA into the optimal threshold, F1 score, precision, and recall. (PDF) [file pone.0262463.s003.pdf]

Case	(1)				(2)				(3)				(4)				Best score			
Time series	Threshold	F_1	Precision	Recall	Threshold	F_1	Precision	Recall	Threshold	F_1	Precision	Recall	Threshold	F_1	Precision	Recall	Threshold	F_1	Precision	Recall
1	27.4	0.67	1.00	0.50	49.1	0.67	1.00	0.50	1.6	0.01	0.01	0.50	-0.2	0.00	0.00	1.00	27.4	0.67	1.00	0.50
2	2.6	0.84	0.87	0.81	19.5	0.97	0.94	1.00	33.7	0.70	0.62	0.81	100.7	0.54	0.39	0.88	19.5	0.97	0.94	1.00
3	7.4	0.77	0.91	0.67	32.4	0.97	1.00	0.93	8.1	0.13	1.00	0.07	4.0	0.77	0.91	0.67	32.4	0.97	1.00	0.93
4	2.7	0.73	0.67	0.80	2.7	0.67	0.75	0.60	4.4	0.29	0.50	0.20	16.0	0.40	0.40	0.40	2.7	0.73	0.67	0.80
5	6.6	0.50	0.50	0.50	2.6	0.80	0.67	1.00	25.9	0.50	0.50	0.50	26.1	0.50	0.50	0.50	2.6	0.80	0.67	1.00
6	1.8	0.75	0.75	0.75	2.8	0.59	0.56	0.63	70.6	0.40	0.43	0.38	11.6	0.60	0.50	0.75	1.8	0.75	0.75	0.75
7	0.9	0.52	0.41	0.71	0.9	0.20	0.12	0.76	48.4	0.13	0.08	0.42	4.6	0.26	0.47	0.18	0.9	0.52	0.41	0.71
8	2.7	0.95	1.00	0.90	NA	NA	NA	NA	2.5	0.02	0.01	0.80	9.2	0.20	0.20	0.20	2.7	0.95	1.00	0.90
9	4.5	0.29	0.33	0.25	7.4	0.33	0.30	0.38	7.0	0.20	0.50	0.13	7.0	0.20	0.50	0.13	7.4	0.33	0.30	0.38
10	5.9	0.27	1.00	0.15	6.0	0.38	1.00	0.23	1.3	0.03	0.02	0.08	12.6	0.14	1.00	0.08	6.0	0.38	1.00	0.23
11	2.0	0.95	0.95	0.95	2.7	0.50	0.62	0.42	4.6	0.44	0.54	0.37	6.3	0.52	0.48	0.58	2.0	0.95	0.95	0.95
12	2.5	0.50	0.50	0.50	NA	NA	NA	NA	2.5	0.00	0.00	0.50	13.6	0.80	0.67	1.00	13.6	0.80	0.67	1.00
13	1.3	0.57	0.80	0.44	1.3	0.57	0.80	0.44	8.0	0.31	0.50	0.22	10.8	0.18	0.50	0.11	1.3	0.57	0.80	0.44
15	1.3	0.50	0.75	0.38	1.6	0.50	0.75	0.38	6.7	0.33	0.50	0.25	84.1	0.20	0.50	0.13	1.3	0.50	0.75	0.38
16	12.2	0.80	1.00	0.67	12.2	0.86	0.75	1.00	4.5	0.80	1.00	0.67	4.4	0.86	0.75	1.00	4.4	0.86	0.75	1.00
17	1.0	0.67	0.71	0.63	1.5	0.81	0.93	0.72	3.9	0.42	0.30	0.70	-1.5	0.39	0.24	0.98	1.5	0.81	0.93	0.72
19	1.1	0.68	0.62	0.75	2.4	0.82	0.91	0.75	-0.4	0.38	0.24	0.99	-0.4	0.41	0.27	0.89	2.4	0.82	0.91	0.75
20	-10.3	0.19	0.15	0.27	1.3	0.11	0.67	0.06	-2.5	0.11	0.07	0.30	-2.5	0.16	0.09	0.82	-10.3	0.19	0.15	0.27
21	2.1	0.57	0.50	0.67	5.5	0.77	0.71	0.83	3.7	0.22	0.17	0.33	11.5	0.29	1.00	0.17	5.5	0.77	0.71	0.83
22	1.0	0.90	0.98	0.83	1.0	0.94	0.94	0.94	-0.9	0.26	0.27	0.25	-0.5	0.22	0.15	0.41	1.0	0.94	0.94	0.94
23	3.2	0.74	0.70	0.78	7.3	0.53	0.50	0.56	28.6	0.84	0.80	0.89	51.7	0.62	0.45	1.00	28.6	0.84	0.80	0.89
24	2.6	0.13	1.00	0.07	2.4	0.45	0.71	0.33	2.9	0.13	1.00	0.07	2.6	0.83	0.86	0.80	2.6	0.83	0.86	0.80
25	2.6	0.99	1.00	0.98	NA	NA	NA	NA	2.1	0.08	0.40	0.05	2.0	0.12	0.38	0.07	2.6	0.99	1.00	0.98
26	0.9	0.14	0.08	0.99	0.7	0.18	0.10	0.99	0.7	0.26	0.19	0.43	2.4	0.27	0.21	0.36	2.4	0.27	0.21	0.36
27	1.9	0.50	0.50	0.50	2.5	0.40	0.33	0.50	2.7	0.08	0.04	0.50	5.8	0.33	0.25	0.50	1.9	0.50	0.50	0.50
28	0.8	0.15	0.08	0.98	0.8	0.19	0.10	0.99	-5.5	0.44	0.49	0.41	2.2	0.33	0.67	0.22	-5.5	0.44	0.49	0.41
29	2.2	0.40	0.50	0.33	2.9	0.62	0.57	0.67	4.1	0.55	0.60	0.50	1.6	0.67	0.67	0.67	1.6	0.67	0.67	0.67
30	9.7	0.48	0.38	0.67	3.5	0.60	0.43	1.00	-78.1	0.02	0.01	0.89	2.1	0.51	0.35	1.00	3.5	0.60	0.43	1.00
31	2.3	0.25	0.18	0.42	NA	NA	NA	NA	2.3	0.10	0.05	0.83	4.4	0.41	0.26	1.00	4.4	0.41	0.26	1.00
32	1.1	0.34	0.27	0.45	7.4	0.46	0.38	0.60	3.4	0.19	0.11	0.70	4.3	0.24	0.16	0.47	7.4	0.46	0.38	0.60
33	18.2	0.67	1.00	0.50	94.5	0.80	0.67	1.00	2.4	0.00	0.00	0.50	13.4	0.80	0.67	1.00	13.4	0.80	0.67	1.00
34	1.6	0.36	0.50	0.29	1.7	0.43	0.43	0.43	6.6	0.36	0.50	0.29	2.5	0.40	0.38	0.43	1.7	0.43	0.43	0.43
36	NA	NA	NA	NA	7.2	0.50	0.33	1.00	NA	NA	NA	NA	2.7	0.50	0.33	1.00	2.7	0.50	0.33	1.00
37	0.9	0.16	0.09	0.65	1.6	0.21	0.21	0.21	-0.1	0.07	0.04	0.97	-0.1	0.07	0.04	0.94	1.6	0.21	0.21	0.21
38	1.4	0.33	0.67	0.22	1.2	0.40	0.31	0.56	2.7	0.57	0.80	0.44	1.1	0.67	0.83	0.56	1.1	0.67	0.83	0.56
39	1.4	0.44	0.50	0.40	2.5	0.53	0.80	0.40	7.2	0.29	0.50	0.20	2.2	0.52	0.41	0.70	2.5	0.53	0.80	0.40
40	0.9	0.14	0.08	0.89	0.7	0.20	0.11	0.98	2.8	0.47	0.38	0.60	2.0	0.44	0.40	0.48	2.8	0.47	0.38	0.60
41	2.6	0.67	1.00	0.50	NA	NA	NA	NA	3.6	0.15	0.09	0.50	NA	NA	NA	NA	2.6	0.67	1.00	0.50
42	2.7	0.76	0.73	0.80	2.7	0.29	0.50	0.20	-1.3	0.09	0.04	0.98	-0.2	0.10	0.05	0.84	2.7	0.76	0.73	0.80
43	4.6	0.23	0.50	0.15	6.8	0.66	0.61	0.70	2.8	0.18	0.43	0.11	7.6	0.73	0.94	0.59	7.6	0.73	0.94	0.59
44	2.1	0.50	1.00	0.33	4.4	0.40	0.50	0.33	25.0	0.50	1.00	0.33	4.3	0.75	0.60	1.00	4.3	0.75	0.60	1.00
45	NA	NA	NA	NA	NA	NA	NA	NA	NA	NA	NA	NA	2.6	0.04	0.02	1.00	2.6	0.04	0.02	1.00

Case	(1)				(2)				(3)				(4)				Best score			
Time series	Threshold	F_1	Precision	Recall	Threshold	F_1	Precision	Recall	Threshold	F_1	Precision	Recall	Threshold	F_1	Precision	Recall	Threshold	F_1	Precision	Recall
46	0.9	0.18	0.10	0.88	0.8	0.24	0.14	1.00	-1.7	0.53	0.57	0.50	2.1	0.54	0.63	0.48	2.1	0.54	0.63	0.48
47	1.3	0.27	0.40	0.20	1.2	0.29	0.50	0.20	1.7	0.47	0.57	0.40	2.7	0.43	0.33	0.60	1.7	0.47	0.57	0.40
50	1.3	0.33	0.40	0.29	1.3	0.31	0.33	0.29	2.9	0.01	0.00	0.29	5.8	0.60	1.00	0.43	5.8	0.60	1.00	0.43
51	1.7	0.33	0.50	0.25	2.2	0.40	1.00	0.25	10.4	0.50	0.50	0.50	10.6	0.57	0.67	0.50	10.6	0.57	0.67	0.50
52	1.8	0.43	0.60	0.33	4.9	0.18	0.50	0.11	2.7	0.57	0.80	0.44	1.1	0.67	0.83	0.56	1.1	0.67	0.83	0.56
53	2.6	0.13	1.00	0.07	2.4	0.45	0.71	0.33	2.9	0.13	1.00	0.07	2.6	0.83	0.86	0.80	2.6	0.83	0.86	0.80
55	1.7	0.44	0.50	0.40	2.3	0.44	0.50	0.40	8.8	0.55	0.50	0.60	17.3	0.60	0.60	0.60	17.3	0.60	0.60	0.60
56	1.7	0.44	0.50	0.40	2.6	0.67	0.75	0.60	4.5	0.55	0.50	0.60	4.4	0.67	0.57	0.80	2.6	0.67	0.75	0.60
57	NA	NA	NA	NA	4.3	0.25	0.20	0.33	0.7	0.00	0.00	0.67	20.8	0.50	1.00	0.33	20.8	0.50	1.00	0.33
58	1.6	0.89	0.95	0.84	19.5	1.00	1.00	1.00	2.2	0.09	1.00	0.05	2.5	0.15	0.40	0.09	19.5	1.00	1.00	1.00
60	134.1	0.47	0.67	0.36	19.0	0.42	0.32	0.64	106.3	0.35	0.50	0.27	42.0	0.52	0.50	0.55	42.0	0.52	0.50	0.55
61	NA	NA	NA	NA	NA	NA	NA	NA	1.4	0.12	0.09	0.17	0.2	0.14	0.08	0.67	0.2	0.14	0.08	0.67
62	20.7	0.50	0.38	0.75	17.4	0.50	0.33	1.00	0.5	0.02	0.01	0.75	8.7	0.40	1.00	0.25	17.4	0.50	0.33	1.00
63	2.1	0.20	0.50	0.13	NA	NA	NA	NA	7.8	0.08	0.06	0.13	2.7	0.42	0.28	0.88	2.7	0.42	0.28	0.88
65	2.5	0.24	0.38	0.18	2.7	0.18	0.13	0.29	8.0	0.05	0.02	0.41	8.0	0.05	0.03	0.29	2.5	0.24	0.38	0.18
66	2.7	0.92	1.00	0.86	NA	NA	NA	NA	NA	NA	NA	NA	8.0	0.09	0.50	0.05	2.7	0.92	1.00	0.86
67	2.5	0.80	0.74	0.87	2.7	0.90	0.82	1.00	2.3	0.23	0.33	0.17	3.0	0.63	0.51	0.83	2.7	0.90	0.82	1.00
