# Supplementary material for: A simple method for unsupervised anomaly detection: An application to Web time series data
Source: PLoS One. 2022 Jan 11;17(1):e0262463. doi: 10.1371/journal.pone.0262463 (PMC8752013; doi:10.1371/journal.pone.0262463)
Supplement: S3 Table — We show the optimal threshold, F1 score, precision, and recall on 5 time series in the realAdExchange data set under each case. If the F1 score is undefined under any k, we enter NA into the optimal threshold, F1 score, precision, and recall. (PDF) [file pone.0262463.s004.pdf]

Case	(1)				(2)				(3)				(4)				Best score			
Time series	Threshold	F_1	Precision	Recall	Threshold	F_1	Precision	Recall	Threshold	F_1	Precision	Recall	Threshold	F_1	Precision	Recall	Threshold	F_1	Precision	Recall
exchange-2_cpm_results	-199.3	0.00	0.00	0.50	31.9	0.33	0.25	0.50	5.8	0.03	0.01	0.50	24.6	0.05	0.03	0.50	31.9	0.33	0.25	0.50
exchange-3_cpc_results	NA	NA	NA	NA	11.1	0.29	0.17	1.00	NA	NA	NA	NA	2.6	0.04	0.02	1.00	11.1	0.29	0.17	1.00
exchange-3_cpm_results	NA	NA	NA	NA	33.4	0.40	0.25	1.00	NA	NA	NA	NA	33.7	0.67	0.50	1.00	33.7	0.67	0.50	1.00
exchange-4_cpc_results	NA	NA	NA	NA	20.0	0.33	0.25	0.50	6.3	0.00	0.00	0.50	11.4	0.40	0.33	0.50	11.4	0.40	0.33	0.50
exchange-4_cpm_results	2.7	0.80	1.00	0.67	2.7	0.15	0.08	0.67	24.1	0.50	1.00	0.33	25.9	0.44	0.33	0.67	2.7	0.80	1.00	0.67
