# Supplementary material for: A simple method for unsupervised anomaly detection: An application to Web time series data
Source: PLoS One. 2022 Jan 11;17(1):e0262463. doi: 10.1371/journal.pone.0262463 (PMC8752013; doi:10.1371/journal.pone.0262463)
Supplement: Attachment — Submitted filename: Response to Reviewers.docx [file pone.0262463.s005.docx]

October 3, 2021

PONE-D-21-21491

[Title] A simple method for unsupervised anomaly detection: An application to Web time series data

[Author] Keisuke Yoshihara, Kei Takahashi

We wish to express our appreciation to the reviewers for their insightful comments on our paper. The comments have helped us significantly improve the paper. Our comments and revisions are as follows.

**Journal Requirements**

(1) Please ensure that your manuscript meets PLOS ONE's style requirements, including those for file naming.
(2) In your Methods section, please include additional information about your real dataset and ensure that you have included a statement specifying whether the collection method complied with the terms and conditions for the website. Please ensure that the links to both datasets are included in both the Data availability statement and the Methods section.

**Our comments & revisions**

(1) We ensure that our manuscript and file naming meet PLOS ONE’s style requirements.
(2) In the Method section, we have included the additional information about the data used and the statement that we comply with the terms and conditions for the website to collect the data. We also have included the links to datasets in both the Data availability statement and the Method section.

**Reviewer #1’s Comments**

(i) The innovation of the article is not strong. It feels that it is an ordinary statistical method. It is abnormal if it is greater than R times the standard deviation?
(ii) In Table 1, Why is our method better than others? What are the advantages? What is the essential difference between these methods? Just because it’s simple?
(iii) How to select R is not clear.
(iv) In application part, in other data sets, it is proved that our method can be used, but it is not compared with other methods, which can not explain the advantages of our method.

**Our comments & revisions**

(i) Thank you for your comment on this point. However, we believe that you may have misunderstood this point. The methodological novelty of our method is that it is applicable to non-stationary time series data, often observed in Web related field, in contrast to the static models. We have added this point in the Introduction section. Moreover, our detection rule is based on the likelihood ratio, not the standard deviation. At time$n$, we independently perform state estimation of OUR and NULL model by the Kalman filter. Then we obtain the log-likelihood of OUR and NULL model. After that, we determine if the observation is anomalous by comparing its ratio with the threshold. We repeat this process up to time$N$. We have added a supplemental explanation on this point in the Procedure subsection.
(ii) Thank you for your comment on this point. We agree that this point requires clarification. The reason is that our method takes into account the specific information that capture the feature of time series data at the modeling stage, e.g. what time it is. In other words, the number of explanatory variables in the model is much larger in our method than others because other methods basically utilize past observations only when predicting future observations. Furthermore, Braei and Wagner(2020) shows similar findings to ours. The property of the data might affect the performance of our method. We have added a discussion on this point in the Evaluation results subsection.
(iii) Thank you for your comment on this point. We agree that this point requires clarification. In the Evaluation section, we compute the optimal threshold based on the ROC curve such that$F_{1}$score is maximized (See the Procedure subsection). Other studies such as Thill et al.(2017) and Minir et al.(2019) take a similar approach. We have added this point in the Procedure subsection. In the Application section, we determine the value of the threshold based on the comparative statics. We have added a discussion on the determination of the value of the threshold in the Application results subsection.
(iv) Thank you for your comment on this point. However, we consider that this may not be necessary because the data in the Application section is unlabeled and we cannot evaluate each method using unlabeled data. We have the Evaluation section to show the advantage of our method.

**Reviewer #2’s Comments**

(a) The proposed method assumes a normal distribution to realize the log likelihood function and compute the ratios (and sequential learning by taking the difference between n and n-1 step). All the simplicity of the method comes from the assumption. However, such strong assumption should result in inaccurate estimation in the real world. Although the authors displayed experimental results using real-world dataset, they needed to discuss more about the problems and failure cases and show more experimental results with various datasets.
(b) Please explain why the simple method can outperform other complex schemes.
(c) It seems to be ad-hoc to choose the threshold (r) during detection. What are the values used in the experiments and how the values can be determined, systematically?

**Our comments & revisions**

(a) Thank you for your comment on this point. We agree that additional experiments are required. We have added an additional experiment in the Evaluation section. We have additionally applied our method to Numenta Anomaly Benchmark (NAB) dataset, a publicly available anomaly detection benchmark released by Numenta, to evaluate our method. We also have added more discussion on the results in the Evaluation results subsection. Moreover, we have added the reason why we adopt the Gaussian assumption in the Method section.
(b) Thank you for your comment on this point. We agree that this point requires clarification. The reason is that our method takes into account the specific information that capture the feature of time series data at the modeling stage, e.g. what time it is. In other words, the number of explanatory variables in the model is much larger in our method than others because other methods basically utilize past observations only when predicting future observations. Furthermore, Braei and Wagner(2020) shows similar findings to ours. The property of the data might affect the performance of our method. We have added a discussion on this point in the Evaluation results subsection.
(c) Thank you for your comment on this point. We agree that this point requires clarification. In the Evaluation section, we compute the optimal threshold based on the ROC curve such that$F_{1}$score is maximized (See the Procedure subsection). Other studies such as Thill et al.(2017) and Munir et al.(2019) take a similar approach. We have added this point in the Procedure subsection. In terms of the threshold values used, we have added the value of optimal threshold for each time series in the S1 and S2 Table. In the Application section, we used$\underline{r}=3.0$that is determined based on the comparative statics. A systematic determination of the threshold is our future task. We have added a discussion on the determination of the threshold in the Application results subsection.
