# Supplementary material for: A simple method for unsupervised anomaly detection: An application to Web time series data
Source: PLoS One. 2022 Jan 11;17(1):e0262463. doi: 10.1371/journal.pone.0262463 (PMC8752013; doi:10.1371/journal.pone.0262463)
Supplement: Attachment — Submitted filename: Response to reviewers.docx [file pone.0262463.s006.docx]

December 11, 2021

PONE-D-21-21491

[Title] A simple method for unsupervised anomaly detection: An application to Web time series data

[Author] Keisuke Yoshihara, Kei Takahashi

We wish to express our appreciation to the reviewer for insightful comments on our paper. The comments have helped us significantly improve the paper. Our comments and revisions are as follows.

For clarity and consistency, we made a minor revision throughout the manuscript based on the major revisions below.

**Reviewer #3’s Comments**

(1) This article lack of novelty. What are the advantages and major contributions of this method? The likelihood is usually used in detection. What is the difference between other methods?
(2) The explanation why the simple method outperforms other complex schemes is not confidence. It is better to give more believable and confident explanations based on data and experience.
(3) The performance may be largely dependent on the distribution and R-value. The normal distribution assumption lacks sufficient evidence from data and experience. Would the method based on other distributions perform better?

**Our comments & revisions**

(1) Thank you for your comment on this point. We have clearly demonstrated this point in the Introduction section. Please check the part in red in the Introduction section.
(2) Thank you for your comment on this point. We agree the necessity of more believable and confident explanations. We have additionally applied our method including the trend component (random walk) only to the Yahoo A1 benchmark to show that including time-series-specific information into the model contributes to improving the performance. Moreover, we have added the figure showing the estimates of the state variables for time series No.36 and verified that each component actually contributes to the prediction. We have added these two points in the Evaluation results subsection. We believe that these revisions support the explanation why the simple method outperforms other complex schemes.
(3) Thank you for your comment on this point. The objective of this study is to detect anomalies quickly and precisely, not to build an accurate time-series generation model. It is possible to use another distribution in generative or prediction model such as fat-tailed one and the construction of a sophisticated generative model with non-Gaussian distributions may lead to accurate anomaly detection. However, employment of such a model sacrifices the speed of anomaly detection and its extension to multiple dimensions, which are important in this study. Furthermore, our model applies the threshold not to Gaussian distribution but to the ratio of log-likelihood that is distributed as non-Gaussian. In order to make this point clearer and avoid confusion for readers, we have added a note to this point in the Method section. To explain this point more precisely, we also have added the point that the ratio of the log-likelihood is not normally distributed and that the expected value of the NULL model is different from that of OUR model. Please see the part in red in the Method section. Finally, because our model is a Bayesian one, it is inconsistent to use the normality test (Shapiro–Wilk etc.) used by frequentists. Therefore, we do not conduct such a test.
